# VLP-based vaccine induces immune control of *Staphylococcus aureus* virulence regulation

**DOI:** 10.1038/s41598-017-00753-0

**Published:** 2017-04-04

**Authors:** Seth M. Daly, Jason A. Joyner, Kathleen D. Triplett, Bradley O. Elmore, Srijana Pokhrel, Kathryn M. Frietze, David S. Peabody, Bryce Chackerian, Pamela R. Hall

**Affiliations:** 1 University of New Mexico College of Pharmacy, Department of Pharmaceutical Sciences, Albuquerque, NM 87131 USA; 2 University of New Mexico School of Medicine, Department of Molecular Genetics and Microbiology, Albuquerque, NM 87131 USA

## Abstract

*Staphylococcus aureus* is the leading cause of skin and soft tissue infections (SSTIs) and mounting antibiotic resistance requires innovative treatment strategies. *S. aureus* uses secreted cyclic autoinducing peptides (AIPs) and the accessory gene regulator (*agr*) operon to coordinate expression of virulence factors required for invasive infection. Of the four *agr* alleles (*agr* types I-IV and corresponding AIPs1-4), *agr* type I isolates are most frequently associated with invasive infection. Cyclization via a thiolactone bond is essential for AIP function; therefore, recognition of the cyclic form of AIP1 may be necessary for antibody-mediated neutralization. However, the small sizes of AIPs and labile thiolactone bond have hindered vaccine development. To overcome this, we used a virus-like particle (VLP) vaccine platform (PP7) for conformationally-restricted presentation of a modified AIP1 amino acid sequence (AIP1S). Vaccination with PP7-AIP1S elicited AIP1-specific antibodies and limited *agr*-activation *in vivo*. Importantly, in a murine SSTI challenge model with a highly virulent *agr* type I *S. aureus* isolate, PP7-AIP1S vaccination reduced pathogenesis and increased bacterial clearance compared to controls, demonstrating vaccine efficacy. Given the contribution of MRSA *agr* type I isolates to human disease, vaccine targeting of AIP1-regulated virulence could have a major clinical impact in the fight against antibiotic resistance.

## Introduction

Between 2000 and 2012, the incidence of skin and soft tissue infections (SSTI) in the USA is estimated to have increased 40%, with treatment expenditures increasing from $4.4 billion to $13.8 billion in 2012 dollars^1^. Among emergency room patients, the majority of SSTIs are caused by *Staphylococcus aureus*, and over half of these isolates are methicillin-resistant (MRSA)^2, 3^. Compared to antibiotic-susceptible strains, MRSA SSTI treatment failure requires added interventions with associated increases in human suffering and medical costs^4^. Given the ongoing antibiotic resistance crisis, the recurrent nature of *S. aureus* SSTI^5^, and the lack of an approved *S. aureus* vaccine to date^6^, there is an urgent need for alternative approaches to combat infections caused by MRSA.

The production of virulence factors required for *S. aureus* SSTI is largely regulated by the accessory gene regulator operon (*agr*)^7, 8^ through a bacterial communication system known as quorum sensing. Induction of *agr* signaling depends upon the accumulation of small, secreted autoinducing peptides (AIPs) to activate a receptor histidine kinase, AgrC, in the bacterial cell membrane^9, 10^. AgrC activation drives downstream production of the effector molecule, RNAIII, which in turn regulates expression of over 200 virulence genes contributing to invasive infection^7^. *S. aureus* isolates express one of four *agr* alleles (*agr*-I to *agr*-IV), with each secreting a unique AIP (AIP1-AIP4) and expressing a corresponding AgrC. Previously, both an anti-AIP4 monoclonal antibody (mAb)^11, 12^ and an AIP4 immunologic mimotope vaccine^13^ showed protection against infection caused by *agr* type IV isolates. However, antibody or vaccine targeting of signaling by *agr* type I isolates, which are most associated with invasive *S. aureus* infection^14, 15^, has not been reported.

**Figure 1:**
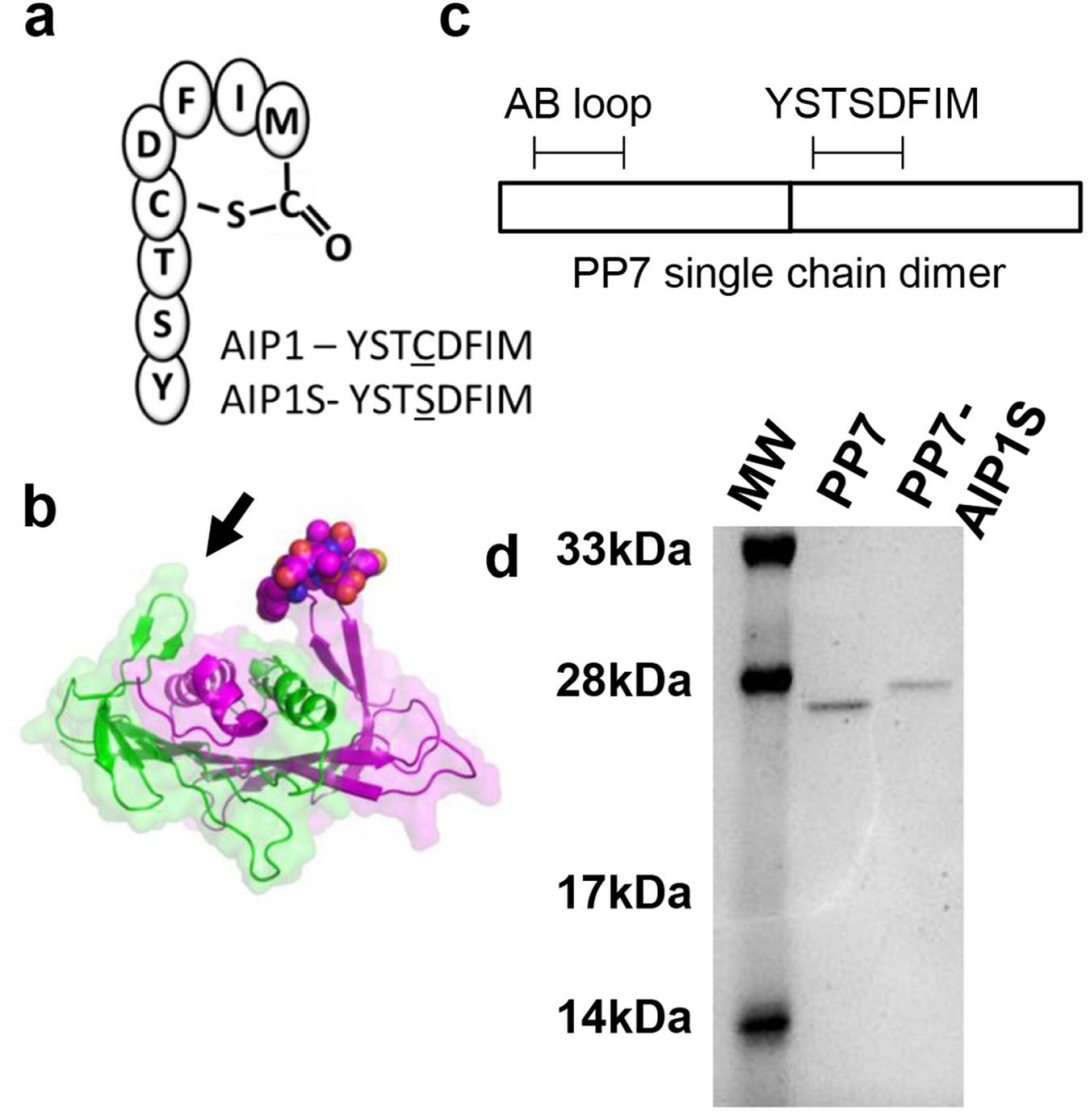
Design and preparation of PP7-AIP1S VLPs. (**a**) Schematic of AIP1 and amino acid sequence of AIP1-C4S (AIP1S). (**b**) Ribbon representation of the PP7 coat protein dimer (one monomer is shown in green and the other in magenta) which can be expressed as a single-chain dimer. Depicted is the first AB loop (indicated by arrow) and the AIP1S sequence (spheres) modeled into the second AB loop (PDB ID 2QUD^21^) using GalaxyWeb^29, 30^. Image prepared using PyMol (PyMOL molecular graphics system, version 1.5.0.4; Schrodinger, LLC). (**c**) Schematic of the site of AIP1S insertion into the second AB loop of the PP7 single chain dimer. (**d**) Coomassie-stained 16% SDS-PAGE showing the relative size of the PP7 single-chain dimer compared to PP7 with the AIP1S insert.

*S. aureus* AIP1 is an eight amino acid peptide (YSTCDFIM) cyclized by a thiolactone bond between the Cys4 side-chain and the carboxyl group of the C-terminal residue (Met8) (Fig. 1a). Given that cyclization is essential for function, immune recognition of the cyclic form of AIP1 may be necessary for antibody-mediated neutralization. However, the small size of these peptides makes them innately non-immunogenic and, together with the labile nature of the thiolactone, increases the difficulty of vaccine development^12, 13, 16^. We sought to overcome these challenges using a bacteriophage virus-like particle (VLP) vaccine platform. These VLPs self-assemble from recombinantly expressed bacteriophage coat proteins which can be genetically altered for surface presentation of practically any epitope in a multivalent format that virtually guarantees strong immunogenicity resulting in high titer, high affinity, and long-lasting antibodies^17^. Specifically, we hypothesized that a vaccine produced by conformationally-restricted presentation of the AIP1 amino acid sequence on the surface of bacteriophage VLPs would elicit antibodies against native AIP1 and induce immune control of *agr* type I-regulated virulence.

To test this, we produced a VLP-based *agr* type I vaccine by cloning a modified AIP1 amino acid sequence (YSTSDFIM) into an immuno-prominent surface loop (the AB-loop) of the *Pseudomonas aeruginosa* RNA bacteriophage PP7 coat protein^18–21^. As expected, the resulting vaccine (PP7-AIP1S) elicited antibodies which recognized AIP1 *in vitro* and was efficacious in a murine SSTI model upon challenge with a highly virulent MRSA *agr* type I isolate. Compared to controls, PP7-AIP1S vaccination resulted in reduced *agr* function and *agr*-regulated virulence factor production at the site of infection. Importantly, PP7-AIP1S vaccination significantly reduced *S. aureus* pathogenesis, based on dermonecrosis and weight loss, and increased bacterial clearance, findings consistent with enhanced host innate defense in the absence of *agr* function^8, 22–26^. Together, these results demonstrate the protective benefits of vaccine-induced immune control of *agr* type I-regulated virulence. Given that several important pathogens utilize similar structurally constrained peptides for virulence regulation^27^, our findings highlight the potential clinical utility of VLP-based vaccines targeting virulence regulators as an alternative or adjunct approach to combat infections caused by other human pathogens.

## Results

### Presentation of the *S. aureus* AIP1 sequence on VLPs induces AIP1-recognizing antibodies

We previously found that vaccination with AIP1 (cyclic or linear) chemically cross-linked to VLPs did not protect mice against subsequent skin infection (unpublished data). This could have been at least in part due to instability of the AIP thiolactone bond^12^ resulting in peptide linearization at some time during the vaccine preparation or vaccination process^13^. Therefore, in an effort to promote immunogenicity and maintain the structural integrity of AIP1 presentation to the adaptive immune system, we inserted a modified AIP1 sequence to be presented in a highly-constrained β-turn on the surface of VLPs assembled from the previously reported PP7 single-chain coat protein dimer^18–20, 28–30^ (Fig. 1a–c). The icosahedral capsid of the *Pseudomonas aeruginosa* RNA bacteriophage PP7 self-assembles from 180 coat protein monomers^31, 32^, whose structural arrangement is equivalent to 90 coat protein dimers^31^. Recombinantly-expressed PP7 coat protein self-assembles into stable VLPs consistent in size with the bacteriophage PP7 icosahedral capsid^28, 31, 33^. The highly constrained β-turn within the PP7 coat protein, called the AB-loop, is displayed on the surface of the assembled capsid^21, 31, 32^ or VLP, and peptides inserted into this loop are highly immunogenic^20, 28, 33, 34^. To avoid potential intermolecular disulfide bond formation via the AIP1 internal cysteine that could negatively impact VLP purification and immune presentation, the inserted AIP1 sequence included a cysteine to serine mutation in position 4 (YSTCDFIM to YSTSDFIM) (referred to as AIP1S) (Fig. 1a). We predicted that this conservative mutation would still allow vaccine induction of AIP1 specific antibodies given (i) that the cysteine side chain is confined to the thiolactone bond in the native molecule, and (ii) that presentation of the AIP1 sequence in the context of the highly constrained PP7 AB-loop would simulate the cyclic nature of AIP1 in the absence of this labile bond. Recombinantly-expressed PP7-AIP1S VLPs resolved into a single peak by size exclusion purification, with agarose gel electrophoresis showing fractions containing a single protein band co-localizing with encapsidated RNA, and with homogeneity shown by dynamic light scattering analysis (Supplemental Fig. S1). Insertion of the AIP1S sequence into the PP7 single-chain coat protein dimer was verified by DNA sequencing of the expression plasmid (data not shown) and by electrophoretic size comparison (Fig. 1d). Given that the equivalent of 90 coat protein dimers are needed to form the PP7 bacteriophage icosahedral capsid, PP7-AIP1S VLPs produced from PP7 single-chain coat protein dimers with the AIP1S insertion in the second AB-loop should therefore display 90 copies of AIP1S for immune stimulation.

**Figure 2:**
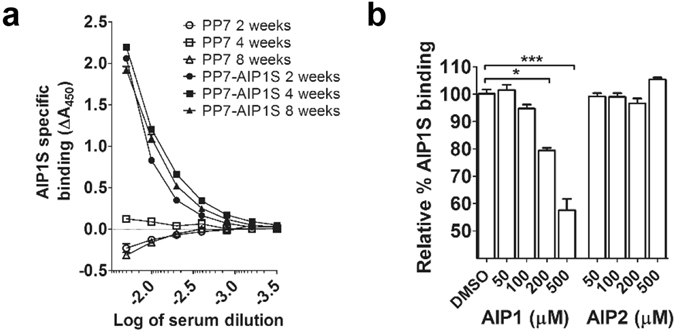
PP7-AIP1S vaccination induces antibodies which recognize soluble AIP1. BALB/c mice were vaccinated twice (i.m.) at 4 week intervals with 10 µg of PP7-AIP1S or PP7 wild-type (control). (**a**) Serum was collected at the indicated time points after the second vaccination. Serum was then pooled (n = 3 mice per group), treated as described in Materials and Methods, and relative binding to PP7-AIP1S determined by ELISA. (**b**) PP7-AIP1S antiserum collected at eight weeks after the second vaccination was prepared as in (**a**), and relative AIP1S binding determined in the presence and absence of the indicated concentrations of AIP1 or AIP2 (n = 3 mice per group; duplicate experiments performed in triplicate). Data are mean ± s.e.m. Kruskal-Wallis ANOVA p < 0.0001 with Dunn’s post-test: *p < 0.05; ***p < 0.001.

We first sought to determine whether vaccination with PP7-AIP1S would induce production of antibodies capable of recognizing *S. aureus* AIP1. To address this, we vaccinated mice with PP7-AIP1S (twice with a 4-week interlude) and then measured the ability of serum antibodies to recognize AIP1. Although prior vaccination efforts using linear or cyclic AIP1 chemically cross-linked to VLPs induced antisera capable of binding immobilized linear or cyclic AIP1 by ELISA (unpublished data), these vaccines failed to provide protection during *S. aureus* infection^13^, possibly suggesting that antibody recognition of the AIP linear tail (Fig. 1a) or linearized AIP is not sufficient for protection. Therefore, here we measured antibody binding to AIP1S as presented in the AB-loop on the surface of PP7, followed by competition binding to soluble, cyclic AIP1. Serum collected at two-, four- and eight-weeks after the last vaccination with PP7-AIP1S, but not after PP7 control vaccination, showed dose-dependent binding to the AIP1S sequence present on PP7-AIP1S VLPs (Fig. 2a and Supplemental Fig. S2). Importantly, in competitive dose-response assays, AIP1S binding by eight-week post-vaccination antiserum (geometric mean titer = 4,550) was inhibited by synthetic cyclic AIP1, but not synthetic AIP2 (GVNACSSLF) (Fig. 2b), demonstrating specificity and the ability to bind native AIP1. Therefore, these results demonstrate that presentation of AIP1S, which lacks the native AIP thiolactone bond, within the PP7 AB-loop is sufficient to elicit antibodies which recognize soluble, native AIP1.

### PP7-AIP1S vaccination provides protection in a murine model of *S. aureus* dermonecrosis

**Figure 3:**
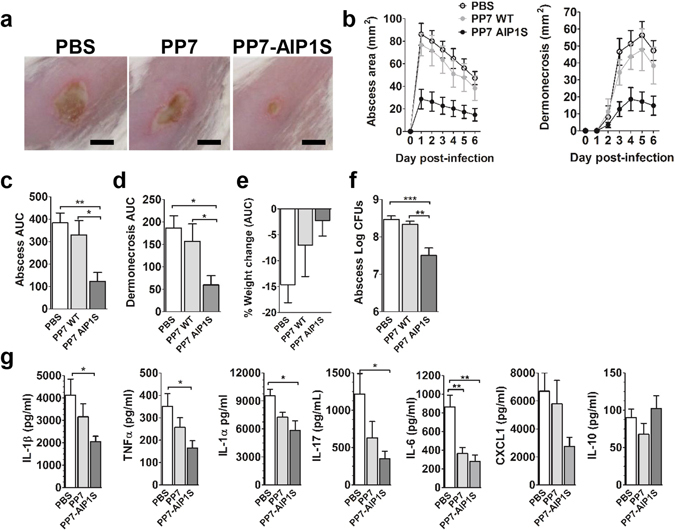
PP7-AIP1S vaccination limits the severity of *S. aureus* skin infection in a mouse model of dermonecrosis. BALB/c mice were vaccinated twice (i.m.) at 4 week intervals with 10 µg of the indicated VLPs or PBS control. Eight weeks after the second vaccination, mice were challenged by subcutaneous infection with 4 × 10^7^ CFU of USA300 LAC. Representative (**a**) day 3 images of infection site and (**b**) daily measures of abscess area and dermonecrosis. Calculated area under the curve (AUC) values for (**c**) abscess area (ANOVA p < 0.0042), (**d**) dermonecrosis (p = 0.0177) and (**e**) percent weight change over the six day infection, as well as (**f**) day 6 bacterial burden at the site of infection (p = 0.0001) (representative of two independent experiments of n = 6 mice per group). (**g**) Cytokine levels in clarified abscess tissue homogenate on day 6 post-infection (ANOVA IL-1β, p = 0.0587; TNFα, p = 0.0358; IL-1α, p = 0.0171; IL-17, p = 0.0322; IL-6, p = 0.0010) (n = 6 mice per group). Data are mean ± s.e.m. Newman-Keuls post test: ns, not significant; *p < 0.05; **p < 0.01; ***p < 0.001.

MRSA isolates of the pulsed-field gel electrophoresis type USA300 (*agr* type I) have long been the cause of most community-associated MRSA (CA-MRSA) infections, and now also cause an increasing number of health-care associated infections^35^. In mouse models of USA300 SSTI, infection with an isogenic *agr*-deletion mutant (Δ*agr*) results in significantly decreased pathogenesis and increased bacterial clearance compared to infection with the wild-type *agr*+ strain^8, 22–25^. Therefore, we postulated that vaccination with PP7-AIP1S would induce immune suppression of *agr*-signaling *in vivo*, thus reducing pathogenesis and increasing bacterial clearance during SSTI. To evaluate the efficacy of PP7-AIP1S vaccination against *agr* type I-mediated virulence and to avoid potential non-specific effects of VLP administration^36^, we challenged mice eight weeks after final vaccination using a well-established mouse model of *S. aureus* SSTI^37^ and the highly virulent USA300 isolate LAC^38^. As expected, PP7-AIP1S vaccinated mice showed reduced abscess formation and dermonecrosis over the course of a six-day infection compared to controls (Fig. 3a–d), although differences in weight loss (used as a measure of morbidity) did not reach statistical significance (Fig. 3e). Importantly, bacterial burden on day 6 post-infection was also significantly reduced in the PP7-AIP1S vaccinated group compared to both PBS and PP7 controls (Fig. 3f). Reduced bacterial burden was consistent with significantly lower local levels of the inflammatory cytokines IL-1β, TNFα, IL-1α and IL-17 in PP7-AIP1S vaccinated mice compared to PBS controls (Fig. 3g). However, differences in local levels of these cytokines between the PP7 WT and PP7-AIP1S groups did not reach significance. In addition, local IL-6 production was significantly reduced in both PP7 WT and PP7-AIP1S vaccinated mice, potentially pointing to a non-specific effect of VLP vaccination on pro-inflammatory cytokine production at this time point. In contrast, significant differences were not observed in local production of CXCL1 or the anti-inflammatory cytokine IL-10. Together, these data demonstrate the efficacy of PP7-AIP1S vaccination against *S. aureus agr* type I-regulated pathogenesis during skin infection.

### PP7-AIP1S vaccination inhibits *S. aureus agr*-signaling *in vivo*

**Figure 4:**
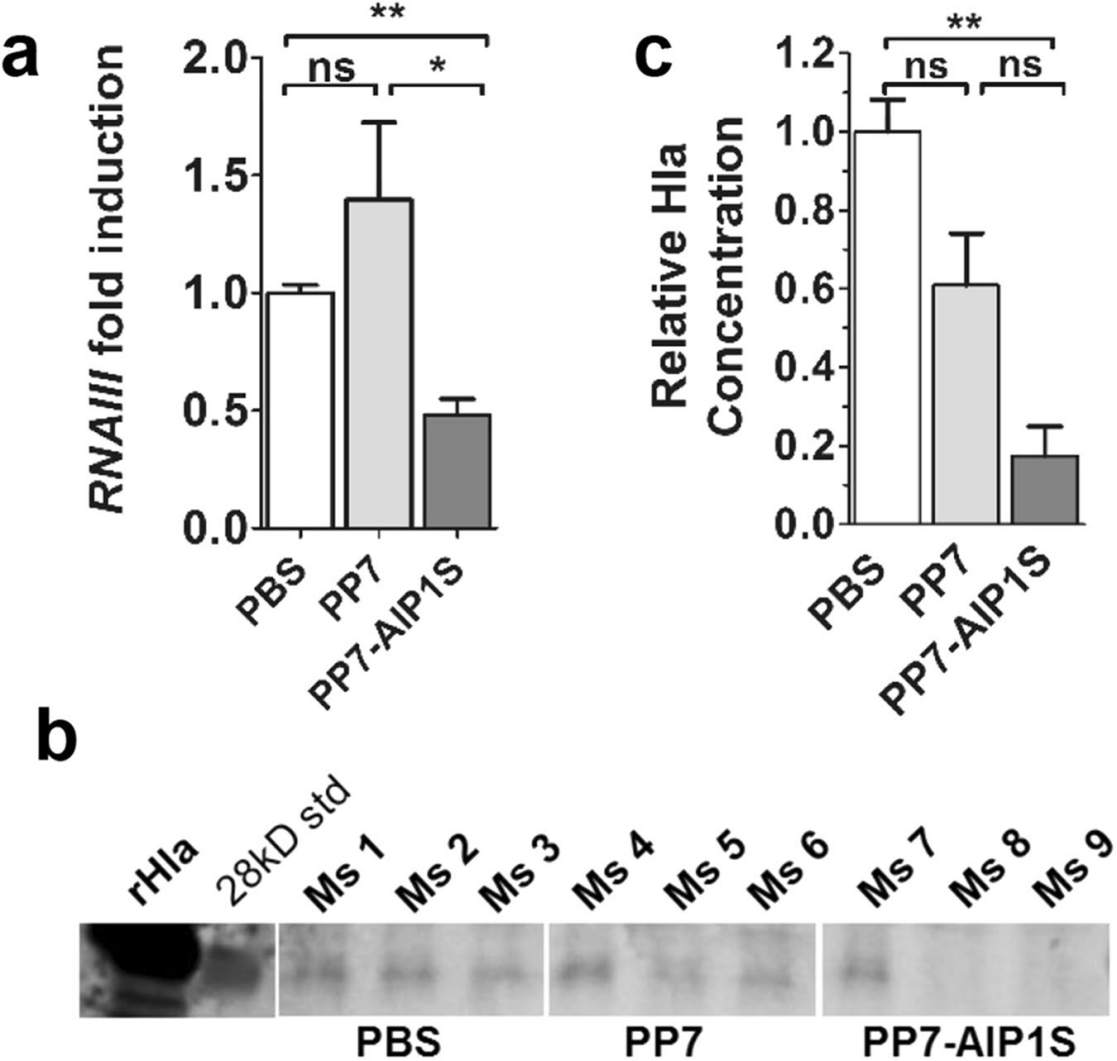
PP7-AIP1S vaccination limits *agr* function at the site of *S. aureus* infection. BALB/c mice were vaccinated twice (i.m.) at 4 week intervals with 10 µg of the indicated VLPs or PBS control. Eight weeks after the second vaccination, mice were challenged by subcutaneous infection with 4 × 10^7^ CFU of USA300 LAC. (**a**) Local RNAIII transcription on day 1 post-infection measured by qPCR (n = 4 mice per group, Kruskal-Wallis ANOVA p = 0.0029). (**b**) Representative immunoblot (showing recombinant Hla, MW marker and 3 mice per group) and (**c**) quantification of Hla levels (relative to PBS control) in clarified abscess tissue homogenate on day 6 post-infection (n = 6 mice per group) (Kruskal-Wallis ANOVA p = 0.0025) with Dunn’s post-test: ns, not significant; *p < 0.05; **p < 0.01.

*S. aureus agr*-signaling induces expression of the effector molecule RNAIII as well as production of alpha-hemolysin (Hla), the causative agent of dermonecrosis^39–43^. The results of our challenge studies, as well as our *in vitro* studies showing that antibodies from PP7-AIP1S vaccinated mice bind soluble AIP1, suggested that vaccination with PP7-AIP1S results in immune suppression of *agr*-signaling during *S. aureus* SSTI. If correct, we would expect reduced RNAIII transcription and Hla expression at the site of infection (local) in PP7-AIP1S vaccinated mice compared to controls. To test this, we measured local RNAIII expression and Hla protein levels on days one and six, respectively, following subcutaneous infection. As expected, RNAIII expression was reduced at the site of infection in PP7-AIP1S vaccinated mice compared to controls (Fig. 4a). Furthermore, day six post-infection Hla levels were reduced in PP7-AIP1S vaccinated mice relative to PBS controls (Fig. 4b,c), although differences between PP7-AIP1S versus PP7 WT vaccination were not statistically significant. Together, these data support a mechanism of action whereby vaccination with PP7-AIP1S induces immune control of *S. aureus agr* type I signaling and virulence regulation during SSTI.

## Discussion

The ongoing antibiotic resistance crisis highlights the urgent need for non-conventional approaches to combat infectious disease, including approaches to inhibit bacterial virulence^44, 45^. In the case of the important human pathogen *Staphylococcus aureus*, virulence regulation is largely mediated by the *agr* operon via secretion of AIPs^9, 10^. These small, conformationally-restrained, secreted peptides bind in an autocrine and paracrine fashion to the bacterial membrane receptor AgrC, which in turn regulates downstream virulence factor expression. Therefore, antibody-mediated sequestration of secreted AIPs could neutralize *agr*-signaling and virulence factor expression on a population level. Of the four *S. aureus agr* types, *agr* type I isolates are most frequently associated with invasive infection^14, 15^. Here we report that multivalent, conformationally-restricted presentation of a modified AIP1 amino acid sequence on VLPs elicits immune control of *S. aureus agr* type I-regulated virulence. Specifically, PP7-AIP1S vaccination (1) induced the production of anti-AIP1 antibodies, (2) limited *agr* type I-signaling *in vivo* and (3) demonstrated efficacy (reduced pathogenesis and increased bacterial clearance) in a mouse model of *S. aureus* SSTI. Given these results and the contribution of *agr* type I isolates to human *S. aureus* infection^14, 15^, vaccine prevention of *agr* type I-mediated virulence could have a major clinical impact and make a significant contribution to the fight against antibiotic resistance.

The diversity of virulence factors produced by *S. aureus* ^7^, many of which disable innate immune cells^46–48^, and the range of infection types (skin, pneumonia, bacteremia, etc.)^7, 49^, suggests that multiple anti-virulence approaches may be needed to limit human disease. For example, targeting specific virulence factors, in particular Hla which is a major contributor to pathogenesis^50^, has shown efficacy in numerous animal models^40, 41, 51–54^ and a monoclonal antibody targeting Hla (MEDI4893) is currently in human clinical trials^55^. Broader approaches aimed at inhibiting *S. aureus* virulence regulation have included peptide and small molecule targeting of the *agr* system^22, 24, 26, 56–68^, as well as development of a monoclonal antibody (mAb) against *S. aureus* AIP4^12, 69^. However, *agr*-signaling has been shown to occur early post-infection and disruption of this early signaling correlates with reduced pathogenesis in the host^22^, suggesting a possible limit to the window of opportunity for therapeutic *agr*-inhibition. Therefore, the development of an efficacious anti-*agr* vaccine could expand the impact of *S. aureus* virulence regulation strategies to have the broadest potential clinical benefit to patients. In this regard, we previously developed a VLP-based AIP4 mimotope vaccine by screening a VLP-peptide library against an anti-AIP4 mAb, AP4-24H11^12, 13, 69^, shown by passive transfer to be protective in a mouse model of *agr* type IV SSTI. Here we advance this work by demonstrating the efficacy of PP7-AIP1S vaccination against *S. aureus agr* type I-regulated virulence using the highly virulent CA-MRSA USA300 isolate LAC^38^. While it will be important in future work to expand these studies to include infections using other *agr* type I isolates versus isolates of heterologous *agr* types, our *in vivo* results, together with *in vitro* data demonstrating that PP7-AIP1S elicited antibodies bind soluble AIP1, but not soluble AIP2, suggest that protection afforded by PP7-AIP1S vaccination is specific to *agr* type-I regulated virulence. Therefore, this VLP-based approach may be utilized to produce a combined vaccine against virulence regulation by each of the *agr* types, thus serving as a valuable component of an overall anti-virulence strategy.

In addition to Staphylococcal species^9, 10^, other human pathogens using *agr*-like quorum sensing systems and secreted peptides to coordinate virulence factor expression^27^ could be targeted by VLP-based vaccination. For example, the food-borne pathogen *Listeria monocytogenes* uses a variety of communication systems to regulate virulence^70, 71^, including an *agr* locus and recently identified secreted AIP^72–76^. In *L. monocytogenes*, the *agr* system regulates over 650 genes contributing to virulence including ones involved in biofilm formation and host cell invasion^73^. Similarly, *Enterococcus faecalis*, an important cause of drug resistant infections^77^, uses the *agr*-like *fsr* gene locus and the secreted, cyclic peptide gelatinase biosynthesis-activating pheromone (GBAP)^78, 79^ to regulate expression of virulence factors important for biofilm formation and pathogenesis^80–85^. Importantly, it has also recently been shown that an *agr* locus regulates production of toxins A and B by the multidrug resistant pathogen *Clostridium difficile* ^86, 87^. These *C. difficile* toxins are directly responsible for disease manifestation^88^ which, in severe cases, can result in sepsis and death^89^, suggesting that interference with *agr*-signaling by this pathogen could significantly limit disease. Therefore, a VLP-vaccine platform could provide a straight-forward approach to elicit immune inhibition of *agr*- and *agr*-like virulence signaling by these and other important human pathogens.

While vaccine targeting of *S. aureus agr*-regulated virulence has clear benefits, potential limitations have also been noted^11, 90, 91^. For example, *in vitro* studies have shown an isolate-dependent association between deletion of *agr* and increased biofilm formation, raising the possibility that inhibiting *agr in vivo* could promote biofilms^92–94^. Increased *in vitro* biofilm formation by *agr* deletion mutants is attributed to increased expression of genes encoding surface associated factors, such as protein A and the fibronectin binding proteins (FnBPs), in parallel with down regulation of secreted virulence factors which promote biofilm dispersal^7, 24, 95–98^. However, *agr*-regulated factors can inhibit phagocyte clearance of *S. aureus* in biofilms^99^, suggesting that targeting *agr in vivo* may also support host innate defense against biofilm infections. A second concern regarding *in vivo* inhibition of *agr* is whether this will select for *agr* mutants. Although most clinical isolates of *S. aureus* are *agr* positive, isolates with mutations in *agr* can arise naturally during chronic infections^15, 100, 101^. However, whether antibody-mediated inhibition of AIP is more likely to select for *agr* mutants than host innate effectors which antagonize *agr*-signaling, such as apolipoprotein B (apoB)^23, 25^, hemoglobin^102^ and reactive oxygen species^103^, has yet to be addressed. Given the contribution of *agr* to *S. aureus* pathogenesis and the protection afforded by vaccine targeting of AIPs, the potential *in vivo* impact of this approach on both *S. aureus* biofilm formation and the selection of *agr* mutants are important areas for future investigation.

Virus-like particles have proven to be a flexible and highly immunogenic platform for vaccine design, and are currently used in FDA-approved vaccines^104^, including Hepatitis B vaccines^105^ and the current nonavalent HPV vaccine (Gardasil 9) designed to induce protection against nine HPV types^106^. Although non-replicating, the dense, repetitive array of coat proteins comprising VLPs is largely unique to microbial antigens and this multivalency triggers a robust immune response in mammals. Therefore, VLPs can dramatically increase the immunogenicity of otherwise poorly immunogenic peptides^17, 107^ including self-antigens^108, 109^. This property, along with the potential for presentation of conformation-dependent antigens, has resulted in investigation of VLP-based vaccines against numerous pathogenic viruses, allergies, cancer, autoimmune disease, Alzheimer’s disease and chronic diseases such as hypertension^17, 110–113^. However, reports of the use of VLP-based vaccines to elicit adaptive immunity against specific bacterial pathogens or proteins have come mainly from our own work^13^ and from research targeting Streptococcal species^114–116^, suggesting that the flexibility of VLP-based vaccine approaches to address bacterial diseases remains largely untapped. Given the FDA approval and success of VLP vaccines against viral pathogens, the use of VLP-based vaccines to prevent infections by the many important human bacterial pathogens warrants further investigation.

In this era of diminishing antibiotic efficacy, a multi-pronged approach, including novel antibiotics, host-targeted therapeutics, vaccines, anti-virulence strategies and combined therapies will likely be crucial for combating disease caused by antibiotic resistant pathogens^11, 44, 45, 117^. Here we present a novel approach to achieve vaccine induced immune control of *S. aureus agr*-regulated virulence. This work highlights the potential clinical utility of VLP-based vaccines as part of an overall strategy to combat infections caused by MRSA and other important antibiotic resistant human pathogens utilizing secreted peptides for virulence regulation^27^.

## Methods

### Ethics statement

Animal studies described herein were approved by the Institutional Animal Care and Use Committee (IACUC) of the University of New Mexico Health Sciences Center (Animal Welfare Assurance number D16-00228) and conducted in strict accordance to recommendations in the *Guide for the Care and Use of Laboratory Animals* ^118^, the Animal Welfare Act, and U.S. federal law.

### Bacterial strains and growth conditions

The CA-MRSA USA300 isolate LAC^38^ (generously provided by Dr. Frank DeLeo, Rocky Mountain National Laboratories, National Institutes of Health, Hamilton, MT) was used for infection studies. Early exponential-phase bacteria were prepared as previously described^119^ and stored at −80 °C for no more than two weeks prior to use. For infection studies, bacteria were diluted in USP-grade saline (B. Braun Medical, Irvine, CA) to yield 4 × 10^7^ CFU per 50 µL. The number of CFU was verified by plating ten-fold serial dilutions onto Trypticase soy agar containing 5% sheep blood (Becton, Dickinson and Company; Franklin Lakes, NJ).

### VLP cloning, expression and purification

The pDSP7K plasmid^120^, encoding the PP7 single-chain dimer under the T7 promoter and transcription terminator, was used for synthesis of PP7-AIP1S VLPs in *E. coli*. With pDSP7K as a template, PCR was used to produce an insert fragment encoding a KpnI restriction site, the modified AIP1 sequence (YSTSDFIM), and a downstream BamHI site (forward primer 5′-GGC GGT ACC TAC AGT ACC TCT GAC TTC ATC ATG GAG GCT ACT CGC ACT CTG ACT GAG-3′; reverse primer 5′-CGG GCT TTG TTA GCA GCC GG-3′). The PCR fragment was inserted into the pDSP7K at the KpnI and BamHI restriction sites and insertion was verified by sequence analysis.

*E. coli* C41 cells (Lucigen, Middleton, WI) transformed with pDSP7K or the pDSP7K-AIP1S expression plasmids were grown at 37 °C to an OD_600_ of 0.8. Expression was induced with 1 mM IPTG, cells cultured for an additional 3 hours, and harvested by centrifugation. Cell pellets were lysed and VLPs purified essentially as described previously^20^ but with size exclusion purification using a 16/60 Sephacryl S-400 HR column (GE Healthcare, Pittsburgh, PA). VLP purity was verified by SDS-PAGE and agarose gel electrophoresis plus Coomassie and ethidium bromide staining. VLPs were concentrated using Amicon Ultra Centrifugal filter units (100 K MWCO) (EMD Millipore, Billerica, MA), and concentrations determined by SDS-PAGE comparison to hen egg lysozyme concentration standards (Sigma-Aldrich, St. Louis, MO). Homogeneity was based on modality analysis using a Malvern Zetasizer Nano Z (Malvern, UK). VLP aliquots were stored at −20 °C until use.

### Mouse immunizations

Four week old, female BALB/cJ mice (Jackson Laboratories, Bar Harbor, ME, USA) were immunized by injection into the caudal thigh muscle with 50 µL of PBS alone or containing 10 µg of either PP7-AIP1S or PP7. Mice received an identical injection four weeks after the initial dose. Serum for ELISA analysis was collected by cardiac puncture at two, four or eight weeks after the second vaccination, with challenge experiments performed at the eight week time-point.

### ELISA

ELISA plates to measure serum antibody binding to AIP1S were prepared by coating Ultra Cruz ELISA High Binding plates (Santa Cruz Biotechnology, Santa Cruz, CA) with 125 ng per well of recombinant PP7 or PP7-AIP1S in 50 µL PBS and incubating 20 hours at room temperature (RT) with shaking. After removing excess liquid, plates were blocked for 2 hours with PBS containing 0.05% Tween-20 and 1% casein. To reduce PP7- and potential *E. coli*-binding antibodies (depleted serum) mouse serum was treated as follows: Serum was diluted 1:50 in PBS and incubated for one hour at RT with end-over-end rotation together with recombinant PP7 (10 µg per 300 µL diluted serum) and PBS-washed C41 cells (the *E. coli* strain used for VLP-expression) (~9 × 10^6^ CFUs). The mixture was centrifuged (5 min at 11,600 × *g*) to remove antibody bound to C41 cells, and the intermediate depleted serum processed through an Amicon Ultra Centrifugal filter unit (100K MWCO) to remove antibody bound to PP7. The presence of antibody in the filtrate (depleted serum) was verified by SDS-PAGE and Coomassie staining. The final depleted serum was serially diluted onto PP7- or PP7-AIP1S-coated ELISA plates and incubated for 1 hour at RT. Murine antibodies bound to VLPs were detected using goat anti-mouse poly-HRP secondary antibody (ThermoFisher Scientific, Waltham, MA) and developed using 1-Step™ Ultra TMB-ELISA according to manufacturer’s directions (ThermoFisher Scientific). For each serum sample and dilution, AIP1S specific binding (ΔA_450_) was equal to the A_450_ for PP7-AIP1S binding minus the A_450_ for PP7 binding. For competition ELISAs, depleted serum was incubated for 1 hour at 37 °C with the indicated concentrations of AIP1 or AIP2 (BioPeptide Co., Inc., San Diego, CA) before addition to PP7-AIP1S-coated ELISA plates.

### Mouse skin infection model

The mouse model of dermonecrosis was implemented essentially as previously described^37^. One to three days before infection (eight weeks after the second vaccination), Nair™ was used to depilate the right flank of the mice (site of infection). On the day of infection, mice were anesthetized by isoflurane inhalation and infected by subcutaneous injection of 50 µL of saline containing 4 × 10^7^ CFU of LAC. Mice were weighed the day of injection and daily thereafter until sacrifice. Injection sites were photographed daily and abscess and dermonecrosis areas determined by analysis with ImageJ^121^. Six days after infection, mice were sacrificed by CO_2_ asphyxiation and a 2.25-cm^2^ section of skin surrounding the abscess was excised for mechanical disruption. Abscess homogenate was serially diluted and plated on sheep blood agar to determine infection site bacterial burden. The remaining homogenate was clarified by centrifugation and the clarified fraction stored at −80 °C until cytokine analysis.

### Cytokine analysis by multiplex assay

Clarified abscess tissue homogenates were quick thawed at 37 °C and concentrations of the indicated cytokines determined using a BioPlex 200 system and BioPlex manager software (Bio-Rad, Hercules, CA) together with a custom-designed mouse multiplex assay (EMD Millipore, Billerica, MA) according to manufacturer’s directions.

### RNA isolation from tissue and quantitative PCR analysis

For analysis of day one post-infection bacterial gene transcription, 2.25 -cm^2^ sections of skin surrounding the infection site were harvested, minced, and stored in RNA*later* (Qiagen, Valencia, CA) at −20 °C. RNA was isolated using QIAzol (Qiagen) and purified using RNeasy kits (Qiagen) according to manufacturer’s directions. cDNA conversion from RNA was performed with a High Capacity cDNA Reverse Transcription Kit (Applied Biosystems, Foster City, CA) and specific primers for *S. aureus* 16S (reverse, 5′-TTC GCT CGA CTT GCA TGT A-3′) or RNAIII (reverse, 5′-GATGTTGTTTACGATAGCTTACATGC-3′) (Integrated DNA Technologies, Coralville, IA). Quantitative PCR (qPCR) was performed using a ViiA-7 RT-PCR system (Applied Biosystems), the specific primers and probes^23^ for 16S (forward primer, 5′-TGA TCC TGG CTC AGG ATG A-3′; reverse primer above and probe 5′-CGC TGG CGG CGT GCC TA-3′) and RNAIII (forward primer, 5′-AAT TAG CAA GTG AGT AAC ATT TGC TAG T-3′; reverse primer above and probe 5′-AGT TAG TTT CCT TGG ACT CAG TGC TAT GTA TTT TTC TT-3′) (Integrated DNA Technologies) and TaqMan Gene Expression Master Mix according to the manufacturer’s protocol (Applied Biosystems). Data are shown as the fold expression of RNAIII versus 16S and relative to the PBS control.

### Tissue Hla quantification by Western blot

For Western blot analysis of Hla levels in clarified abscess homogenate, frozen samples were quick thawed and equal amounts of total protein (based on A_280_) were electrophoresed on 16% Tris-glycine SDS-PAGE gels (Life Technologies, Grand Island, NY). Following transfer to polyvinylidene fluoride membrane, membranes were blocked overnight at 4 °C with TBST (20 mM Tris, pH 7.5, 150 mM NaCl, 0.1% Tween 20) with 5% nonfat dry milk. Hla was detected using sheep anti-Hla primary antibody (ab15948, Abcam, Cambridge, MA) and alkaline phosphatase-conjugated rabbit polyclonal anti-sheep secondary. Membranes were developed with nitroblue tetrazolium (NBT)/5-bromo-4-chloro-3-indolyl-phosphate (BCIP) (Thermo Scientific). Band intensity relative to recombinant Hla control was measured on a FluorChem R system using AlphaView software (ProteinSimple, San Jose, CA).

### Statistical analysis

GraphPad Prism version 5.04 (GraphPad Software, San Diego California) was used for all statistical evaluations. One-way ANOVA parameters followed Bartlett’s test for equal variances and were used with Bonferroni’s (ANOVA) or Dunn’s (Kruskal-Wallis test, non-parametrics) post-hoc multiple comparison analyses. Results were considered statistically significant at p < 0.05.

## Electronic supplementary material

Supplementary Data
