# Supplementary material for: VLP-based vaccine induces immune control of Staphylococcus aureus virulence regulation
Source: Sci Rep. 2017 Apr 4;7:637. doi: 10.1038/s41598-017-00753-0 (PMC5429642; doi:10.1038/s41598-017-00753-0)
Supplement: Supplementary file 1 — Supplementary Data [file 41598_2017_753_MOESM1_ESM.pdf]

SUPPLEMENTARY DATA

VLP-based vaccine induces immune control of *Staphylococcus aureus* virulence regulation

Seth M. Daly[1,+], Jason A. Joyner[1,+], Kathleen D. Triplett[1], Bradley O. Elmore[1], Srijana Pokhrel[1], Kathryn M. Frietze[2], David S. Peabody[2], Bryce Chackerian[2] and Pamela R. Hall[1,*]

[1]University of New Mexico College of Pharmacy, Department of Pharmaceutical Sciences, Albuquerque, NM 87131, USA

[2]University of New Mexico School of Medicine, Department of Molecular Genetics and Microbiology, Albuquerque, NM 87131, USA

*phall@salud.unm.edu

[+]these authors contributed equally to this work

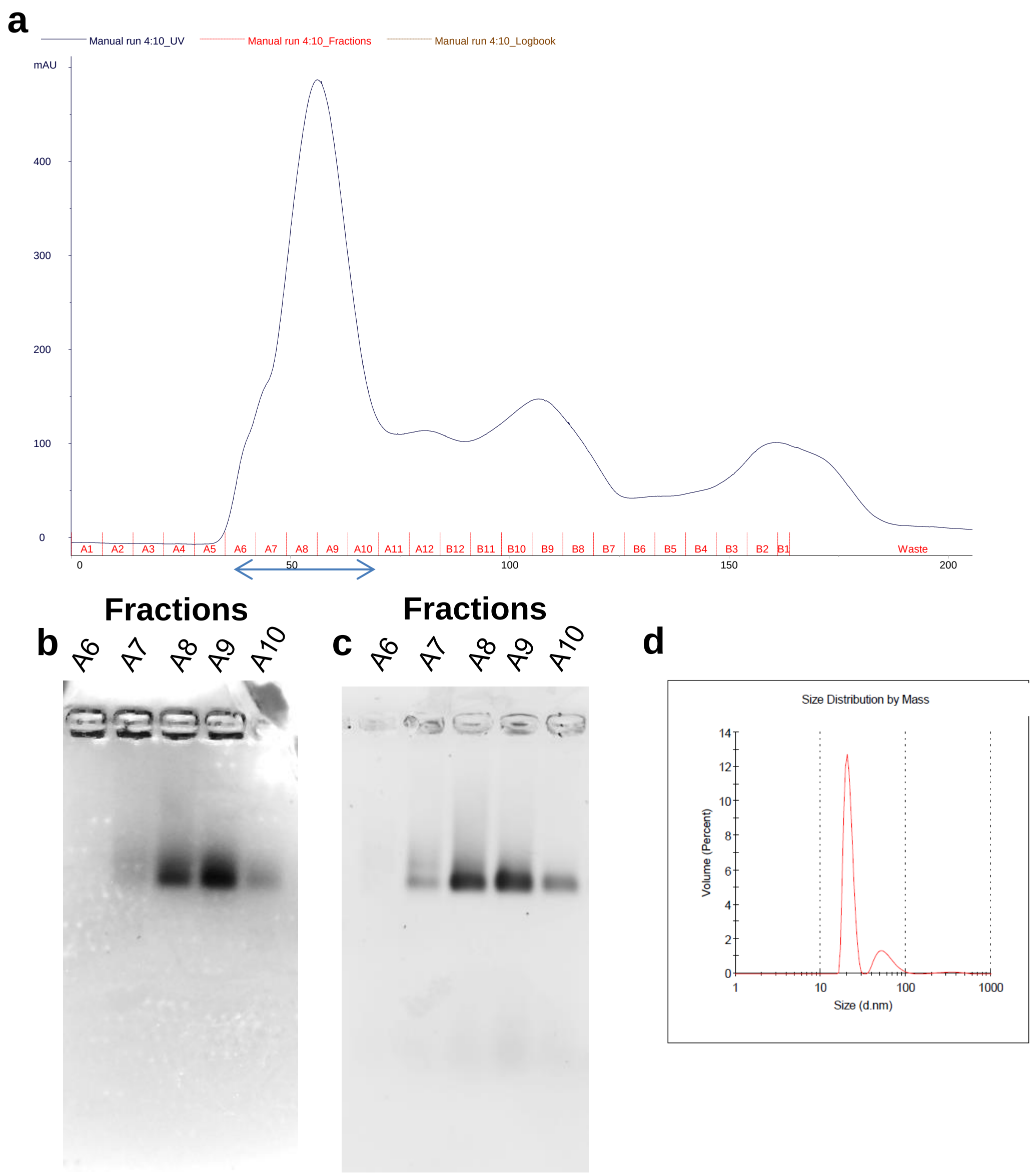

Supplementary Figure S1. Purification of PP7-AIP1S VLPs. (**a**) Representative size exclusion purification of PP7-AIP1S VLPs using a 16/60 Sephacryl S-400 HR column (chromatogram produced using UNICORN software, GE Healthcare, Pittsburgh, PA). Arrow indicates fractions analyzed by agarose gel electrophoresis in (b) and (c). (**b**) Coomassie (protein) staining and (**c**) ethidium bromide (EtBr) staining showing VLP-encapsulated nucleic acid. (**d**) Dynamic light scattering analysis of purified PP7-AIP1S in PBS (Malvern Zetasizer Nano Z, Malvern, UK) showing a major peak consistent in size with PP7 VLPs and a higher diameter peak consistent with PP7 aggregation (reduced with low concentration detergent).

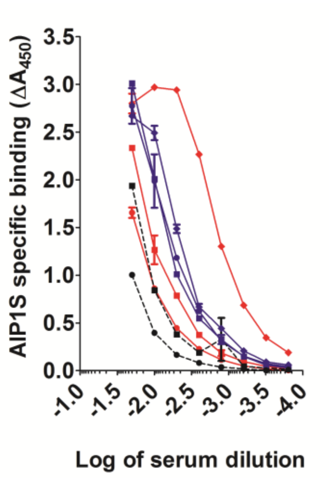

Supplementary Figure S2. AIP1S-specific binding by antisera from individual PP7-AIP1S vaccinated mice. Binding to AIP1S by sera from individual mice is shown in black (dashed lines) (2 weeks), blue (4 weeks) and red (8 weeks). Data are mean ± s.e.m. Analyses of pooled sera from these groups of mice and from groups of PP7 vaccinated mice are shown in Figure 2.
